# Open grid model of Australia’s National Electricity Market allowing backtesting against historic data

**DOI:** 10.1038/sdata.2018.203

**Published:** 2018-10-23

**Authors:** Aleksis Xenophon, David Hill

**Affiliations:** 1 The University of Hong Kong, Pokfulam, Hong Kong; 2 The University of Sydney, Sydney, Australia

**Keywords:** Energy grids and networks, Energy economics

## Abstract

Rising electricity prices, concerns regarding system security, and emissions reduction are central to an energy policy debate under way in Australia. To better evaluate mechanisms that seek to address the nexus of engineering, economic, and environmental challenges facing the country’s electricity system, we have constructed network and generator datasets describing the operation of Australia’s largest transmission network. These data have been collated using open-source software, and are available under an open license. They include the geospatial locations of network elements, and have been designed to interface with a public database maintained by the Australian Energy Market Operator. This interface allows historic data, such as generator dispatch and regional load signals, to be integrated with market models. Interactive network maps, independent datasets, and power-flow models have been used to assess the completeness and functionality of the derived datasets. In the context of Australia, these data can be used to examine geospatial and temporal impacts of power injections from renewables. More generally, they allow market models to be benchmarked against realised outcomes.

## Background & Summary

Australia’s largest transmission network is located along the country’s eastern seaboard, delivering power to 90% of the population^1^. Generators and loads within this network participate in the National Electricity Market (NEM), with the Australian Energy Market Operator (AEMO) overseeing system operation. In recent years, the rapid deployment of intermittent renewable generators within some NEM regions has coincided with the closure of several baseload power plants^2^. This shift has presented a number of engineering and economic challenges, which have been compounded by a complex energy policy landscape that exists within the country. Federal Government initiatives, such as the Renewable Energy Target^3^, incentivise investment in renewables but do not consider the impacts of intermittency associated with these technologies. Following a major blackout in South Australia in 2016, new policies have been proposed at state and federal levels that seek to reward dispatchable forms of generation^4,5^. Concurrently, there is a long-running debate regarding the future direction of climate change policies within Australia, with numerous schemes proposed over the past 10 years^6–8^. The cacophony of mechanisms with competing objectives has created an uncertain environment for investors, with industry experts describing the situation as a ‘crisis’^9^.

Given the NEM’s significance in the context of Australia’s energy system, modelling this network would provide a powerful means by which to evaluate the expected operation of the network under different policy scenarios. At present, publicly available models of the NEM often ignore topological considerations^10^, or greatly simplify the network using a transshipment approach^11^. Research initiatives instigated by the Commonwealth Scientific and Industrial Research Organisation have attempted to address this issue by considering the NEM’s topology in the context of predicting future electricity prices, emissions, and system security^12,13^. Models used in these studies, along with other works analysing similar issues^14–16^, typically use^17^ as the basis for their network data, which has been designed to capture the long, linear structure of the transmission network along Australia’s eastern seaboard, and is comprised of 14 generators, 45 buses, and 55 AC transmissions lines. However, this network is only “loosely based on the southern and eastern Australian networks”^17^, and was developed with the intention to investigate small-signal stability issues. While AEMO may provide more comprehensive models to market participants, and in some cases researchers^18^, they are made available on a confidential basis. Consequently, there is considerable scope for the development of an open grid representation of the NEM.

Open grid models generally require network elements to be manually geocoded based on diagrams provided by the system operator^19,20^, or make use of open-licensed geospatial datasets which contain network information^21^. To-date, such models within the literature typically focus on European grids, with no geospatial representations developed for Australian networks. Despite the absence of such a representation, the components required for an open grid model are readily accessible. Data released by AEMO^22^ and Geoscience Australia (GA)^23–25^ provide a substantial amount of information regarding the NEM’s participants and topology. This data descriptor demonstrates how information from these sources can be compiled to produce generator and network datasets that can be used in power-flow studies.

**Figure 1:**
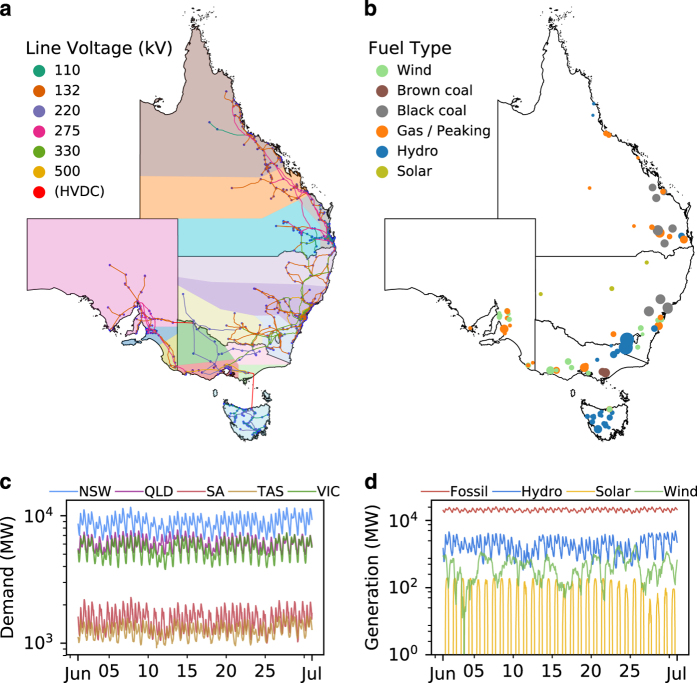
Dataset overview. (**a**) NEM network with transmission lines categorised by voltage level. Coloured areas represent NEM zones. (**b**) Generators by fuel type. Marker size is indicative of relative capacity. (**c**) Demand data at 30 min intervals for each NEM region. (**d**) Aggregate dispatch from fossil, hydro, solar, and wind power plants at 30 min intervals.

Datasets describing generator and network information were constructed independently and then linked via the use of cross-reference tables and database join operations. This modular approach to their design greatly increases their re-use potential and the ease with which they can be updated. In total, the resulting generator dataset contains technical and economic information relating to 203 generating units, while the network dataset consists of 912 nodes, 1406 AC edges, and three HVDC links, with line voltages in the range of 110 kV to 500 kV. These datasets have also been designed to easily integrate with AEMO’s Market Management System Data Model (MMSDM) database^26^, which contains public tables describing regional load information, along with dispatch profiles for individual units. To the authors’ knowledge, AEMO is the only system operator to publish such profiles for units participating within an electricity market. This interface also allows market models to be tested and benchmarked against observed outcomes. An overview of the network’s topology, the geospatial distribution of generating assets, regional demand signals, and aggregate generation by fuel type is given in Fig. 1. The following sections describe the methods used to construct these datasets, the process implemented to integrate them, and finally evidence of their technical validity.

## Methods

**Figure 2:**
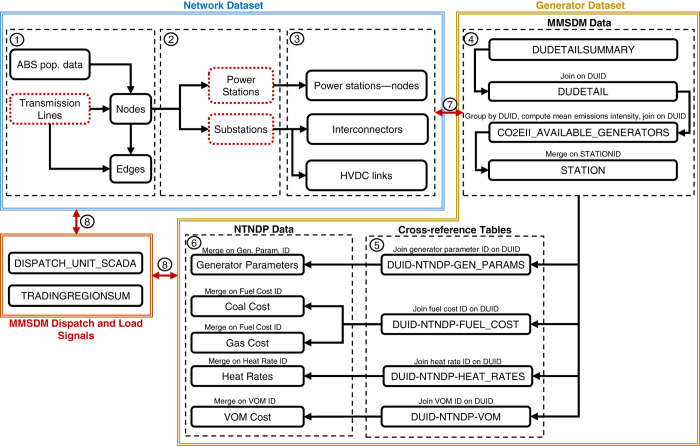
Overview of dataset construction procedures. Red dashed boxes denote GA datasets. (1) Define nodes using coordinates from the ends of transmission lines. Assign ‘from’ and ‘to’ nodes to each line to construct network edges. Use ABS population data to approximate the geospatial distribution of electricity consumption. (2) Assign nodes to power stations and substations based on proximity. (3) Define locations of interconnectors/HVDC links using substation names, and create a map describing power station-node assignments. (4) Use MMSDM tables as the basis for the generator dataset. (5) Join cross-reference table foreign keys to DUIDs. (6) Join generator technical and economic parameters from NTNDP database. (7) Integrate network and generator datasets. Assign DUIDs to network nodes. (8) Integrate unit dispatch and regional load data with network and generator datasets.

The Python programming language was used when constructing both network and generator datasets, with open-source packages, Pandas^27^ and GeoPandas^28^, used extensively when collating and processing GA and AEMO data. An overview of the construction procedure is shown in Fig. 2. The network and generator datasets were integrated by assigning generators to nodes via the use of cross-reference tables. Population data from the Australian Bureau of Statistics (ABS) were also used in conjunction with the network dataset to approximate the geopsatial distribution of electricity consumption. Load and nodal power injection signals from intermittent sources obtained from AEMO’s MMSDM database were then combined with the network dataset, resulting in a geospatial representation of the NEM that can be used in market models. The following sections describe the construction of these datasets, the design of cross-reference tables used to link them, and also the methods implemented to extract historic load and unit dispatch profiles from AEMO’s MMSDM database.

### Network

Information regarding the locations and attributes of major power stations, substations, and transmission lines within Australia were obtained from GA. Metadata accompanying each dataset describes how GA analysts have used satellite imagery in conjunction with high-voltage network diagrams produced by AEMO^29^ when compiling network feature information^30–32^. For each dataset, four files formats are available for download: xls, csv, gdb, and kmz. Of these file types only gdb and kmz files contain coordinates describing the paths of transmission lines. As calculation of line electrical properties is dependent on line length, the choice of suitable file formats was limited to gdb and kmz. For this analysis kmz files were used as, when extracted, they can be interrogated using standard text editors. However, a limitation of using kmz files is the inability to directly load these data into GeoPandas GeoDataFrames - the principle data structure used when handling geospatial network information in this analysis. The construction of GeoDataFrames first required that each kmz file be extracted, yielding a kml file, with the kml file then converted to geojson format using an open-source Python tool, kml2geojson^33^. These geojson files were then imported into GeoDataFrames.

While GA’s datasets are extensive, there are several important limitations associated with their use. The first relates to a lack of information regarding network elements that provide reactive power support to the grid e.g. capacitor banks. Additionally, no information is provided regarding transformers, with this analysis assuming that all transformers have nominal turns ratios, and that there are no phase-shifting transformers. Finally, no information is provided that links network elements between datasets. For example, no details are given linking substations or power stations to transmission lines. Relationships between these elements form the foundation of any network model, which the network construction procedure ultimately seeks to establish.

An overview of the network dataset construction procedure is given in the top-left corner of Fig. 2. Following the filtering of network elements, network nodes and edges were defined. Power stations and substations were then assigned to nodes based on proximity - linking the three GA datasets. Points of connection for HVDC links and interconnectors were identified using substation names. Electrical parameters were then computed for AC edges.

### Filter network elements

Records in the transmission lines, substations, and power stations GeoDataFrames were first filtered, with network elements outside NEM states removed. As this analysis is primarily concerned with the bulk transmission of electrical energy, low and medium voltage lines were also removed - only transmission lines with line-to-line voltages 100 kV and above were retained.

### Construct network nodes

The definition of transmission lines within metadata accompanying GA’s transmission line dataset^30^ informed the strategy used to construct the set of network nodes. GA defines transmission lines as being: “unbroken from end-point to end-point; where end-points coincide with and are snapped to the location of a substation or power station”^30^. Based on this definition, the first and last latitude-longitude coordinate pairs were obtained for each transmission line. Duplicated coordinates were removed, with those remaining describing the locations of all nodes in the network. Node identifiers (IDs) were then assigned to these coordinates.

To link the network model to AEMO’s MMSDM database, and also provide a means of implementing useful aggregation operations when constructing mathematical models of the NEM, additional geospatial information was derived for each node. First, NEM region IDs, which correspond to states within Australia, were assigned to each node (note that AEMO aggregates New South Wales and the Australian Capital Territory into one region). These regions were assigned by using a dataset published by the ABS detailing state and territory boundaries^34^. Spatial join operations were performed to assign a state name to each node based on its geographic location. Region IDs were then assigned to each node based on its state name.

NEM zone IDs were also assigned to each node. These zones describe boundaries at a more disaggregated level than NEM regions, and are used in AEMO’s own market models^35^. While information regarding the exact boundaries of these zones is not made publicly available, diagrams released by AEMO^35^ do provide an approximate description of these zones, along with the locations of some high-voltage transmissions lines. Using transmission lines as a common reference, the diagram produced by AEMO was compared to a map of GA transmission lines, allowing zone boundaries to be geocoded. Fig. 1a illustrates these zones using different colours. Spatial join operations were then performed to assign zones to nodes.

### Construct network edges

The construction of network edges required that relationships be established between the ends of each transmission line and nodes in the network. Note that node locations are given by the set of geographic coordinates describing the ends of transmission lines, with this set filtered such that duplicates were removed. Nodes were assigned to transmission lines by taking the first and last coordinates of each line’s path, and looking up the node IDs that corresponded to these coordinates. The first and last coordinates in a line’s path were defined to be ‘from’ and ‘to’ nodes respectively. Having established a set of nodes and edges, the SciPy^36^ Python package was used to identify disconnected nodes within the grid. In most instances disconnected nodes were located in rural areas, often near mines, and are not a part of the NEM. Few instances of nodes in urban areas disconnected from the greater network were observed. In total, 1.7% of transmission lines and 5% of nodes within NEM regions were removed as a result of this procedure. Total lengths of remaining transmissions lines by voltage level are given in Table 1.

### Assign power stations and substations to nodes

Substations and power station records were assigned to nodes in the filtered and connected network, with these assignments based on proximity. For each substation and power station, the haversine distance between it and all nodes in the connected network was computed. The node closest to the network element, and distance to this node in kilometres, was then returned. In the majority of cases power stations and substations were co-incident with their assigned nodes - consistent with GA’s definition of transmission lines. However, there were instances where this distance was non-zero. This often occurred for wind farms, as turbines can be distributed over large areas, causing these network elements to be located some distance from their nearest node. There were also network elements located in rural areas, often near mines, that were located in NEM states but not part of the NEM network. To filter out these elements a cut-off distance was defined such that a power station or substation could be no more than 100 km from its nearest node. This relatively large cut-off distance has been used as several power stations were observed to be connected to the greater network via transmission lines with voltages less than 100 kV. As these lines were removed when constructing network edges, power stations connected to these transmission lines were located some distance from their nearest node. A distance of 100 km was deemed to provide a suitable trade-off between retaining network elements that were close, but not co-incident, to their assigned node, and removing elements that were clearly disconnected from the network. If this distance was exceeded, the network element was flagged as being disconnected, and omitted from the final dataset.

### Construct HVDC links

Three HVDC branches exist within the NEM. Names of the substations to which they are connected were obtained from AEMO^37^, and looked-up in GA’s substations dataset. Note that in the previous step each substation was assigned a node in the connected network. Using these assignments, ‘from’ and ‘to’ nodes were obtained for each HVDC link. Inspection of GA’s transmission line dataset revealed that HVDC lines were included in the dataset, but no attributes were assigned to differentiate them from AC transmission lines. Using the points of connection defined for each HVDC link, GA transmission lines corresponding to HVDC links were identified and tagged accordingly.

### Compute electrical parameters for AC edges

Having defined the network’s topology, the electrical properties of AC edges were then computed. As data relating to the electrical parameters of transmission lines used in the NEM’s network are scant, they have been derived from first principles using assumptions regarding conductor types and spacing between phases. A public document released by Ausgrid, one of Australia’s largest energy companies, suggests that Pluto AAC 19/3 conductors are often used in their new medium and high-voltage transmission lines^38^. The derivation of electrical parameters for transmission lines assumed that all conductors within the network are of this type, with conductor properties obtained from manufacturer specifications^39^. The geometric mean radius and per-length resistance of this conductor, along with the assumed system base power and frequency are listed in Table 2. With respect to the geometric configuration of transmission lines, it has been assumed that phases are equally spaced, with minimum separation distances between phases for lines at different voltage levels, *D*_*min*_, obtained from^40^. In practice it is likely that a safety factor is applied to this minimum separation distance. In the absence of additional information, a safety factor of 1.5 is adopted, which is multiplied by the minimum separation distance to yield the assumed distance between phases, *D*_*eq*_. Using these data the inductance, *L*, inductive reactance, *X*_*L*_, line-neutral capacitance, *C*_*N*_, and shunt susceptance, *B*_*N*_, were calculated. Base impedance, *Z*_*base*_, and base admittance values, *Y*_*base*_, are also computed for lines at each voltage level, *V*. These parameters are presented in Table 3, which also includes the formulae obtained from^41^ used in their derivation. The length of each transmission line, *l*, was then computed by summing the haversine distance between adjacent points constituting each line’s path. Nominal per-unit values for each AC edge were calculated by multiplying the length of each transmission line by per-unit per-length electrical parameters corresponding to its voltage level. It should be noted that the simplifying assumptions adopted ignore the effects of conductor bundling, coupling between phases and neutral conductors, and coupling between parallel lines. For this reason the code used to construct the network dataset, available at^42^, has been designed to easily accommodate different electrical parameters, which can be updated according to user requirements.

### Generator dataset

An up-to-date repository of generator information can be found within AEMO’s MMSDM database, accessible via the nemweb portal^43^. Datasets within nemweb differ in content, and also the frequency with which they are updated. To accommodate the data requirements of market participants, each month AEMO aggregates MMSDM data, sending this information to participants via DVDs. In 2017 AEMO began releasing public versions of these Particpant DVDs^22^. The release of these data significantly increases the ease with which generator information can be accessed, reducing the effort required to sort, store, and clean MMSDM data. Registered capacities of generators, ramp-rates, regional demand, and historic generator dispatch can be found within these tables. As the schema of this database is time invariant, DVDs from different time periods can be used interchangeably, with the possibility to combine data across multiple months to perform long-run analyses. At the time of writing, data from July 2009 to December 2017 are available for download.

Basic technical parameters for units, such as registered capacities, were obtained from MMSDM tables. Unit specific parameters relating to the economics of plant operation, such as fuel costs, start-up costs, and minimum up and down times, were obtained from AEMO’s National Transmission Network Development Plan (NTNDP)^44^ database. An important difference between these two sources is the frequency with which they are released. MMSDM Participant DVDs are released monthly, while components of the NTNDP database are typically updated annually. Construction of the generator dataset involved the systematic compilation of information from these two sources. First, this procedure involved constructing a list of all generators within the NEM. As MMSDM data are updated monthly, an up-to-date list can be found using these tables. It should be noted that MMSDM tables provide details for generators at differing levels of aggregation. At the most disaggregated level, AEMO identifies collections of prime movers as a genset. A collection of gensets constitutes a Dispatchable Unit Identifier (DUID), and a collection of DUIDs constitutes a station. Inspection of MMSDM tables revealed that DUIDs are used when reporting historic generator output, which motivated the decision to use DUIDs as primary keys in the compiled generator dataset. Doing so establishes a link between the compiled generator dataset and historic dispatch for each generator, which can then be easily integrated into market models - a novel attribute of this dataset.

While MMSDM tables use a consistent convention when referring to generators, the same is not true for tables within the NTNDP database. Often units are identified by DUIDs, but in some cases different conventions apply. In order to join NTNDP and MMSDM data, cross-reference tables were manually constructed. These tables map generator IDs in NTNDP tables to DUIDs in MMSDM tables. If the underlying datasets are updated or changed, only the cross-reference tables need to be updated. There were instances where corresponding NTNDP IDs could not be found for some DUIDs. Where possible, an existing unit of similar type in the NTNDP database was assigned to the DUID. Assumptions made when making these assignments were documented in the comments field within each cross-reference table.

In this analysis MMSDM tables for June 2017 have been used to construct the generator dataset. This time period was selected for two reasons. First, this period is after the retirement of Hazelwood power station in March 2017, which was one of Australia’s largest baseload generators. As we anticipate that these datasets will have future-oriented applications, we seek to use a time period after this plant had ceased operation, as this prevents dispatch signals for other DUIDs being influenced by output from Hazelwood. The second reason for selecting this time period relates to the availability of AEMO datasets which can be used to cross-check registered capacities listed in MMSDM tables. AEMO periodically publishes such datasets, with one available for June 2017^45^. By using MMSDM data from the same period, registered capacities of generators in different regions can be cross-checked against an additional source.

Database operations performed to construct the generator dataset are illustrated on the right-side of Fig. 2. Each box represents a table within the underlying MMSDM and NTNDP datasets, with the database operation used to join each table to the compiled dataset displayed above each box. The direction of arrows denotes the order in which each operation is performed. The collation procedure started by taking an MMSDM table providing a summary of DUID details as the basis. This table also contained a version history of records for each DUID. As we wish to extract the most recent records, a filter was applied to remove records corresponding to previous versions. Registered capacities, station names, and emissions intensities were then joined to this basis table using DUIDs as join keys. It should be noted that additional steps were required when joining emissions intensities to the compiled dataset as they are reported by genset. Each emissions intensity record contains the genset ID, and also the DUID corresponding to the genset. A group-by operation was performed to compute the mean emissions intensity for each collection of gensets which constituted a DUID. In most cases gensets within these collections had identical emissions intensities, but some instances were observed where emissions intensities differed among gensets. As it is impossible to determine the proportion each genset contributes to aggregate output for each DUID, computation of the mean emissions intensity was deemed to provide a suitable trade-off, making the implicit assumption that each genset contributes equally to DUID output. It should also be noted that this emissions intensity table was obtained from the Current Reports directory on nemweb^46^, as participant DVDs do not provide information allowing emissions intensities to be mapped to DUIDs.

Having compiled MMSDM data, foreign keys from cross-reference tables were joined to this table using DUIDs as join keys. These foreign keys were then used to join data from each NTNDP table to the compiled dataset. Note that the arrows for joining cross-reference tables are in parallel, illustrating that these join operations can be performed in any order. Finally, two attributes were assigned to each DUID. First, a fuel category was assigned based on the specific fuel-type associated with each DUID. All coal and natural gas power plants were assigned the fuel category ‘Fossil’. Hydro, wind, solar, and plants using biofuels were assigned the categories ‘Hydro’, ‘Wind’, ‘Solar’, and ‘Biofuel’ respectively. Mapping from fuel-types to general fuel categories assisted in performing aggregation operations, allowing units of different types to be easily identified and grouped. The short-run marginal cost (SRMC) of electricity production for each generator was then computed based on each unit’s heat rate, fuel cost, and variable operations and maintenance (VOM) cost, as shown in equation (1), with fuel costs for 2016–17 used in the calculation. There was one gas-fired power station, Swanbank E, for which a 2016–17 fuel cost is unavailable. This unit’s SRMC was based on its 2017–18 fuel cost instead.

(1)SRMC[$/MWh]=HeatRate[GJ/MWh]×FuelCost[$/GJ]+VOMCost[$/MWh]

While generally comprehensive for thermal power plants, generators with the following primary fuel types: natural gas (pipeline), coal seam methane, kerosene - non-aviation, and diesel oil had no minimum dispatchable output assigned. In practice there would be a level below which output from these generators would non-dispatchable. As a number of these power plants are peaking facilities, minimal online production levels are likely to be dependent on plant specific characteristics. Given the difficulty associated with their approximation, we do not attempt to estimate these values. Instead we acknowledge the incompleteness of these records, and suggest users be mindful of this omission in any potential use cases. Additionally, the NTNDP database does not contain economic parameters for several hydro generators. As these power plants do not have fuel costs, the SRMC is equal to their VOM cost. It was observed that hydro generators in the NTNDP database were assigned a VOM cost of 7 $/MWh, irrespective of their capacity. Consequently, for hydro generators missing economic parameters, a default SRMC of 7 $/MWh was assigned. Similarly, several wind and solar generators were missing VOM costs. Inspection of the NTNDP database revealed that these generators were always assigned a VOM cost of 0 $/MWh, with this value set as the default SRMC for wind and solar plants missing economic parameters. Technical parameters were also missing for solar power plants. Ramp-rates for these generators were instead obtained from MMSDM tables reporting maximum ramp-rates for DUIDs. Note that these differ from those obtained from the NTNDP database, which report ramp-rates for start-up, shut-down, and normal operation. As power output is variable for wind and solar generators, it was assumed that there is no required minimum on or off period for these units. Start-up costs for solar generators were also assumed to be zero.

### Geospatial load distribution

AEMO provides load-signals for each NEM region at the temporal resolution of 30 min intervals. However, no information is provided regarding the spatial distribution of load with regions. In order to allocate load to nodes, a similar approach to that used by^19,20^ was implemented. This load assignment strategy assumes that the geospatial distribution of load follows that of population - areas with larger populations are assumed to consume proportionally more electricity. To implement this strategy, geospatial population data were obtained from the ABS^47^. It should be noted that these data are available at differing levels of aggregation, with each geographic boundary referred to as a Statistical Area. In this analysis Statistical Area Level 2 (SA2) population datasets were used, as boundaries at this level of aggregation seek to “represent a community that interacts together socially and economically”^48^. The dataset is comprised of 1875 areas which are within states and territories that constitute the NEM, with the population in 2015 given for each record. An approximation of the population served by each node was made by constructing a Voronoi tessellation of the network, using network nodes as seeds, and then overlaying Voronoi cells with SA2 areas. The steps taken to compute the population within each Voronoi cell, and by extension the population served by each node, are given in Algorithm 1. For each Voronoi cell the SA2 areas with which it intersects were found. The proportion of an SA2 area’s population living within each intersected area was estimated by computing the area of intersection as a fraction of total SA2 area, and multiplying this value by the SA2 area’s population. Population contributions from each intersected SA2 area were then summed, yielding an estimate of the total population within each Voronoi cell, and by extension the population served by the node within that cell. The total population assigned to each node was then divided by the total population of the NEM region in which the node was located. The resulting fraction is the proportion of the NEM region’s population assigned to that node, which was used as a proxy for the proportion of regional electricity consumed at that node.


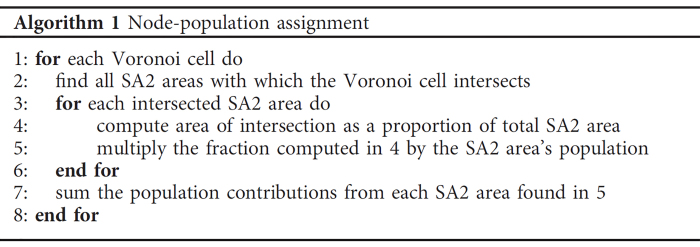


**Figure 3:**
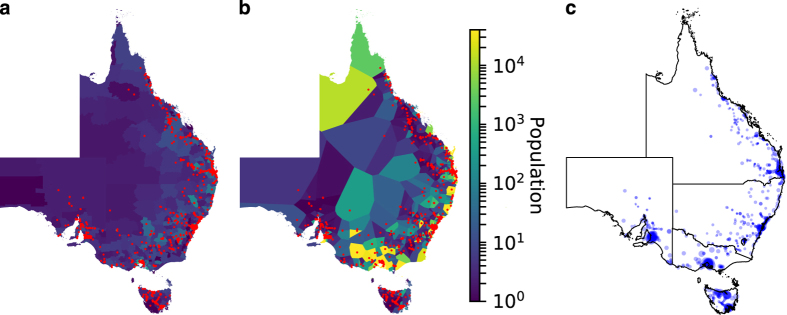
Demand allocation. (**a**) SA2 areas with the locations of network nodes denoted by red markers. (**b**) Voronoi tessellation with colour denoting population in each polygon. (**c**) Final demand allocation. Size of dots indicates proportion of regional demand allocated to node.

Figure 3 illustrates the data that underlies the demand allocation procedure, with Fig. 3a showing SA2 areas, while Fig. 3b illustrates the Voronoi tessellation based on network nodes. Fig. 3c shows the final demand allocation with dot sizes representative of the proportion of regional demand consumed. From this figure it can be seen that more electricity is consumed near each capital city - a pattern of demand consistent with expectations.

### Linking datasets

#### Generator and network datasets

The generator and network datasets developed in the previous sections have been constructed independently, with this modular approach to their design simplifying the process of applying updates. In order to make use of these datasets when constructing market models, they must be integrated. This was accomplished by creating a map between DUIDs in the generator dataset and nodes in the network dataset. This involved mapping DUIDs to GA power stations, with these power stations having already been mapped to nodes in the network construction phase. The schematic below illustrates the DUID-node assignment procedure.


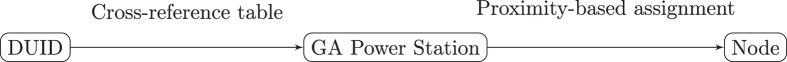


For some DUIDs a corresponding GA power station could not be found. The locations of these DUIDs were manually looked-up, and compared against a map of GA power stations. If an alternative GA power station was located close to a DUID, this station was assigned instead. Note the different functions of the projections described in the above schematic. Assigning DUIDs to GA power stations essentially attributes geospatial information to DUIDs. The second map then projects power stations onto the set of network nodes, capturing the geospatial distribution of generating assets. Therefore, direct correspondence between DUIDs and GA power stations is not necessary, what is important is the ability of the assigned GA power stations to approximate the locations of DUIDs. This assignment strategy is particularly advantageous when one considers making alterations to the network’s topology. If for example transmission lines were filtered by a different voltage level, a new set of nodes would arise. GA power stations would again be assigned to nodes based on proximity. The static cross-reference table would still assign DUIDs to the same GA power stations, allowing DUIDs to be assigned to the new set of nodes. Consequently, this allocation procedure allows generator and network datasets to be easily integrated following an alteration to the network’s topology.

#### MMSDM load profiles

Load profiles for each node were constructed by linking tables from AEMO’s MMSDM database to the network dataset previously described. Demand profiles are provided for each NEM region within the MMSDM database, with demand given at 30 minute intervals. For each interval, the proportion of regional demand consumed at each node was multiplied by the level of regional demand corresponding to that node. This yielded nominal demand profiles for each node in MW. As MMSDM table schema do not change over time, demand profiles for any period between July 2009 and December 2017 can be constructed. In this analysis we have extracted and transformed regional load profiles for the month of June 2017, which can be found in signals_regional_load.csv (1).

#### Historic dispatch

AEMO’s MMSDM database contains historic dispatch signals for each DUID, with output in MW given at 5 min intervals. To keep demand and dispatch signals at the same level of temporal resolution, these dispatch data were aggregated, yielding mean power output at 30 minute intervals for each DUID, and can be found in signals_dispatch.csv (1). When developing models, these data were used to construct signals for intermittent nodal power injections. This was accomplished by first identifying DUIDs corresponding to intermittent generators. These intermittent generator DUIDs were then grouped by the node to which they were assigned, with their corresponding dispatch profiles superimposed. The resulting profiles take into account the spatio-temporal relationship of nodal power injections from intermittent sources. Mathematical models within the GitHub repository associated with this project^42^ contain code implementing the signal construction procedure described here. In addition to constructing signals for intermittent generators, these data also provide a unique opportunity to compare historic dispatch to results obtained from market models. Due to data availability issues, it has previously been impossible to benchmark such models against realised market outcomes. However, dispatch profiles released by AEMO now make such analyses possible.

Similar to the load data described above, MMSDM table schema for dispatch profiles are time invariant. As the MMSDM database is periodically updated we have not attempted to construct long-run generator dispatch profiles, as they would soon be outdated. Instead we have included signals for the month of June 2017 as a sample, and made available a tool that can be used to extract dispatch and load signals for any month within the MMSDM database, which can be found at^42^.

### Code availability

All code used to construct the network and generator datasets is contained within two Jupyter Notebooks at the GitHub repository associated with this project^42^. Data that underlies these datasets, along with notes relating to their provenance, are also included. Interested parties can interrogate each step of the dataset construction process and re-produce all results. A third notebook provides a tool used to extract generator and dispatch load signals from MMSDM tables. Additional Jupyter Notebooks are provided which contain code used in the data validation process, and implement market models using the generator and network datasets as inputs. A Jupyter Notebook that plots all results from these analyses is also included, allowing output from the network construction and data validation procedures to be visualised. All code is available under an MIT license.

## Data Records

Eight csv files constitute the network and generator datasets: generators.csv, network_edges.csv, network_nodes.csv, network_hvdc_links.csv, network_ac_interconnector_links.csv,

network_ac_interconnector_flow_limits.csv, signals_dispatch.csv, and signals_regional_load.csv (1). Tables 4 and 5 summarise the inputs used to construct the network and generator datasets respectively. An overview of fields within datasets describing network nodes, edges, HVDC links, and interconnectors are presented in Tables 6, 7, 8, 9, 10. Fields within the generator dataset are described in Table 11, while Tables 12 and 13 describe fields in regional load and DUID dispatch time series. Note that we have not elected to use the IEEE Common Data Format^49^ for this dataset. Several reasons motivated this decision. First, the standard format does not support the inclusion of historic dispatch and regional load signals. Secondly, it does not support the inclusion of geospatial information, such as latitude and longitude coordinates, which have been provided for network nodes. Thirdly, AEMO’s definition of interconnector limits, obtained from^50^, are incompatible with this format. While most interconnector limits are with respect to the flow over a single link, the Victoria to New South Wales interconnector is defined as the aggregate flow over several lines. For this reason interconnector information has been split into two files: network_ac_interconnector_links.csv which contains the nodes to which each AC interconnector is attached, while network_ac_interconnector_flow_limits.csv contains the aggregate bi-directional flow limits for each AC interconnector. HVDC link information is also contained within a separate file, with this file structure enabling these records to be more easily examined, maintained, and updated.

The files provided at 1 provide a complete description of the final network and generator datasets. Those seeking to re-produce these datasets can do so by using Jupyter Notebooks within the GitHub repository^42^ associated with this project.

## Technical Validation

Validation of the derived datasets seeks to assess their completeness, consistency, and functionality. Here completeness and consistency refers to the extent to which the derived data capture information relating to features within the network, while functionality assesses the ability to use these data when formulating mathematical representations of the NEM. When assessing completeness, where possible, derived data have been compared to additional datasets, not used in their derivation, to independently verify their contents. Graphical techniques have also been used to construct interactive network maps which assisted in verifying the logical consistency of relationships between elements. With respect to functionality, the ability to construct feasible mathematical models was first verified. Historic dispatch and interconnector flow signals were then used as a benchmark against which the results from generic power-flow models were compared. It should be noted that there are several limitations associated with using a modelling approach to validate these datasets. Considerations such as contractual obligations between generators and retailers, strategic bidding behaviour of generators, and hydro scheduling constraints have not be taken into account. Unplanned outages and crew availability are additional factors that could cause model and observed outcomes to diverge. It is therefore not expected that dispatch and interconnector flows will exactly correspond. Rather the goal is to determine if qualitative trends associated with the basic economic and operational aspects of the NEM can be captured using these data. In this way the following assays seek to provide an overall assessment regarding the capability of the presented datasets to emulate the operation of the system which they seek to represent.

### Completeness and consistency

#### Generator data

Information within the generator dataset was first evaluated by examining the number of DUIDs within MMSDM tables that were assigned to network nodes, and therefore included in the final generator dataset. It was noted that MMSDM tables contain more unique DUIDs than the final generator dataset; however, not all of these correspond to physical units. For example, DUIDs pre-fixed with ‘RT’ or ‘DG’, acronyms for reserve trader and dummy generator respectively, are in fact components used in the automated dispatch protocol within AEMO’s National Electricity Market Dispatch Engine. These non-generator DUIDs were excluded when comparing DUIDs in the underlying and final datasets. In total 203 DUIDs are present within the final generator dataset, accounting for all scheduled and semi-scheduled DUIDs in the filtered MMSDM tables. Note that non-scheduled units are present in MMSDM tables, but have not been included in the final generator dataset, as these generators do not participate in central dispatch.

Registered capacities were also checked against an additional dataset provided by AEMO. Periodically, AEMO makes available separate datasets containing generation information data detailing registered capacities of generators in each NEM region^45^. Table 14 shows registered capacities for scheduled and semi-scheduled generators for both datasets. The largest difference was observed for installed hydro capacity in New South Wales and Victoria. This is due to Murray power station, a hydro power plant with a registered capacity of 1500 MW, being assigned to Victoria in the AEMO generation information dataset, but New South Wales in the compiled generator dataset. Further investigation revealed that this station is in fact located in New South Wales, close to the border with Victoria. Following hydro generators, the next largest discrepancy was associated with installed wind capacity. Differences of 27 and 8% were observed for New South Wales and South Australia respectively, and are due to DUIDs being assigned to wind farms still under construction. For the remaining generator types the difference in installed capacity is less than 5%. In aggregate, less than a 1% difference is observed in installed capacity between datasets, supporting the notion that the compiled generator dataset comprehensively covers generators within the NEM.

While construction of the generator dataset primarily used database join and merge operations, the compilation of data from different sources also relied on manually constructed cross-reference tables mapping DUIDs to foreign keys in NTNDP tables. The template used to create these maps was designed such that several checks could be carried out when assigning NTNDP keys to DUIDs. Fields including station name, registered capacity, and fuel-type were added to the template for each DUID, with this additional information used to ensure that NTNDP keys were being correctly assigned to their corresponding DUID. A comment field was also added to each table, and used to document assumptions made during the assignment process.

#### Network data

An interactive map was created to assess the dataset resulting from the network construction procedure. Produced using Folium^51^, an open-source Python package, the map includes zoom functionality, allowing different sections of the network to be examined in detail. When clicked, nodes display the names and IDs of network elements (substations and power stations) which were assigned to them. Similarly, transmission lines when clicked display the line’s name, along with it’s GeoDataFrame ID. This map provided a useful way in which to visually inspect different network areas, and assess if any errors had occurred during the network construction process. It also provided a useful means by which to quickly identify individual nodes within the network, for example those connected to HVDC links or interconnectors. The interactive map generated by this code can be found within this project’s GitHub repository^42^.

#### Demand data

Owing to a lack of disaggregated demand data it has not been possible to validate computed values for the proportion of regional electricity demand consumed at each node. Qualitatively, Fig. 3c demonstrates how the demand allocation procedure results in greater demand within the vicinity of capital cities. While this pattern of demand follows intuition, it has not been possible to verify empirically.

### Functionality

Assessment of dataset functionality involved the development of two baseline mathematical representations of the NEM, both seeking to determine dispatch via a linear cost minimisation objective. The first implements a DCOPF model, based on the formulation presented in^52^, that makes use of the full network comprised of 912 nodes, 1406 AC edges and three HVDC links. The second uses a 16 node transshipment model, with each node corresponding to a NEM zone. This reduced network was constructed by grouping nodes and generators by the zone to which they were assigned. Links between zones were found by mapping zones to ‘from’ and ‘to’ nodes in network_edges.csv and network_hvdc_links.csv, keeping links that connect zones, and dropping duplicates. The ability to quickly construct a reduced network model can be useful if a less granular model is required. It also provides a means by which to compare the informational content of the full network representation with a less detailed model that neglects features such as transmission line electrical parameters and node voltage angles. For both representations a nominal fee of 5$/MWh was imposed on absolute interconnector flows, reflecting the fact that their is economic value associated with using these network assets. The fee is set lower than the SRMC of hydro generators to avoid this penalty distorting dispatch decisions, but still discourages unrealistic loop flows between regions. Pyomo^53,54^ was used to formulate the baseline models, which were solved for each of the 1440 trading intervals within the signals_regional_load.csv dataset using Gurobi^55^ as the solver. Feasible solutions were found for each trading interval, indicating that supply is sufficient to meet demand in all periods. Having established that feasible models can be constructed, three scenarios were developed that make use of historic signals for interconnector flows and generator dispatch in order to assess the ability of the datasets to emulate NEM operation. These scenarios involved modifying the baseline DCOPF and transshipment models, with each scenario run using one day’s worth of data (48 trading intervals). While data for the entire month could be used, the time required to complete simulation runs proved prohibitive. In the case of the baseline DCOPF model, approximately 10 s was required to obtain a solution for each trading interval, requiring a runtime of approximately 4 h for each scenario if using data for all available time periods. By using a single day, the diurnal cycle of load and intermittent power injection profiles can be captured, while reducing the amount of time required to analyse each scenario. The following sections describe the different scenarios constructed, and how they have been used to assess the capability of the presented datasets to emulate NEM operation.

### Scenario 1 - Minimise difference between model and observed outcomes

Scenario 1 is based on the network validation procedure used in^19^, where generator output was adjusted such that interconnector flows obtained from a power-flow model closely matched observed flows. Data for historic flows over each of the network’s six interconnectors were obtained from^43^, providing one example of an additional table that can be included from AEMO’s MMSDM database. A limitation of the method used in^19^ is that it is unknown if the dispatch schedule that minimises interconnector flow deviations is congruent with reality. In this analysis generator dispatch is known, with these data used to extend the validation procedure used in^19^. In addition to minimising the difference between model and observed interconnector flows for each interconnector, deviations between model and observed dispatch are also minimised for each generator. Rather than minimising these deviations via manual adjustment, as in^19^, a mathematical approach is adopted, whereby the objective function given by equation (2) is minimised, where *g* and *i* denote generators and interconnectors respectively.

(2)min∑i‖Model flowi-Observed flowi‖1+∑g‖Modeldispatchg-Observeddispatchg‖1

Large deviations between model and observed interconnector flows could be indicative of incorrectly specified interconnector limits, transmission line electrical parameters, or demand allocations. Similarly, large deviations between observed and model dispatch may indicate incorrectly specified generator parameters.

### Scenario 2 - Fixed nodal injections

Scenario 2 complements Scenario 1 by considering the situation where all generator output is fixed to historic levels. Interconnector flows arising from the model are then compared to observed historic flows. Unlike Scenario 1 where deviations were minimised by design, this scenario attempts to assess how interconnector flow limits, tranmissions line electrical parameters, and demand allocations influence flows throughout the network, and how much these flows deviate from observed outcomes. As historic data for fixed power injections exceeded total demand (due to losses within the real system), it was necessary to scale generator output such that total demand and supply balanced. Without this re-balancing models flows, which do not consider losses, would be infeasible.

### Scenario 3 - Generation cost minimisation

In this scenario generator output was allowed to be variable, with dispatch determined by generator SRMCs and network constraints. Model dispatch and interconnector flows were compared with observed values. Assuming the market for power is somewhat competitive, it is expected that the relative costs of generators will influence their dispatch. By comparing model and observed dispatch, it is possible to ascertain how well generator SRMCs capture these relative cost differences, and reflect the bidding behaviour of generators.

As some unit specific parameters included in the generator dataset, such as minimum up times and startup costs, cannot be analysed using the linear baseline models, an extension is made by formulating a unit commitment model based on^56^ which takes these parameters into account. Minimum reserve levels for each region are also included, and based on data obtained from^44^. The increased computational complexity of this formulation resulted in computer memory limitations being encountered when running the model using the full network. Consequently, results have only been obtained when using the reduced network. By comparing dispatch results from the linear and non-linear formulations, an indication is given as to the effect of including these additional parameters.

### Scenario results

**Figure 4:**
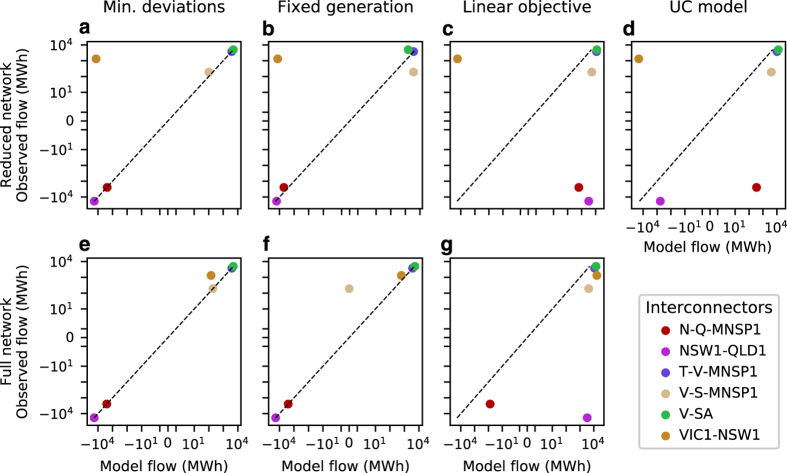
Comparison between model and observed interconnector flows. (**a** & **e**) Scenario 1, minimise difference between model and observed interconnector flows and generator dispatch using the full and reduced networks respectively. (**b** & **f**) Scenario 2, fixed generator output. (**c** & **g**) Scenario 3, variable generator output using a linear cost minimisation objective. (**d**) Scenario 3, variable generator output using a non-linear unit commitment (UC) formulation.

**Figure 5:**
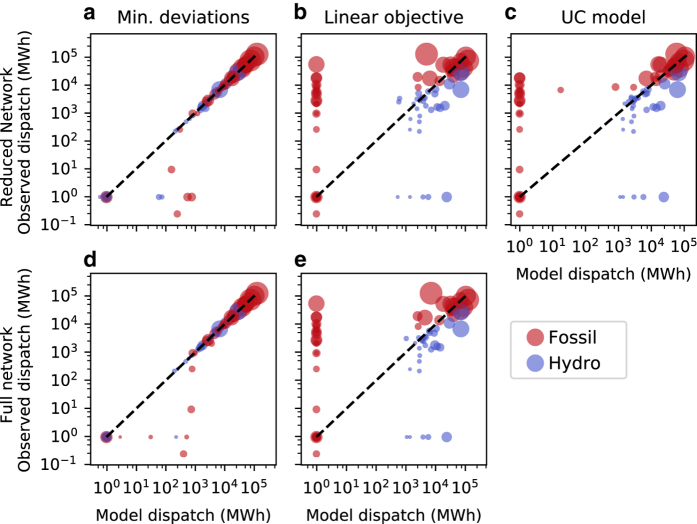
Comparison between model and observed aggregate dispatch. (**a** & **d**) Scenario 1, minimise difference between model and observed interconnector flows and generator dispatch using the full and reduced networks respectively. (**b** & **e**) Scenario 3, dispatch when using a linear cost minimisiation objective for the reduced and full network models respectively. (**c**) Scenario 3, non-linear UC formulation that takes into account temporal constraints, using the reduced network.

**Figure 6:**
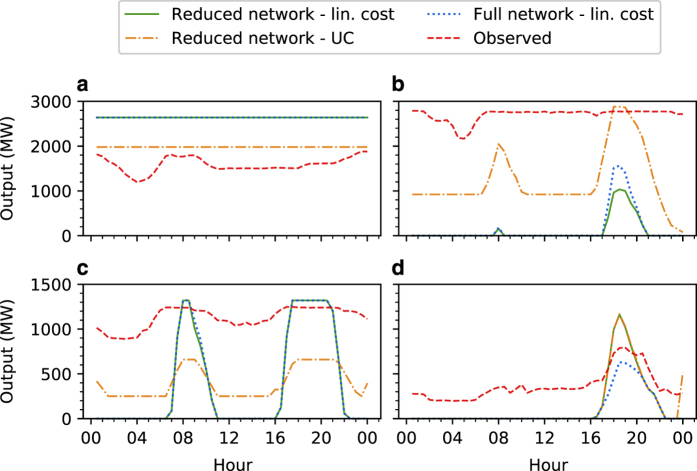
Dispatch profile comparison. (**a**) Bayswater power station. (**b**) Eraring power station. (**c**) Vales Point B power station. (**d**) Torrens Island power station. Green lines denote dispatch from the baseline model using a linear generation cost minimisation objective and reduced network representation. Dashed blue lines denote dispatch arising from a DCOPF model using the full network representation and a linear generation cost minimisation objective. Dashed orange lines denote output from the UC formulation, while dashed red lines denote historic output.

Results from the assays performed are shown in Figs. 4, 5, and 6. Figure 4 compares model and observed net aggregate interconnector flows, while Fig. 5 compares model and observed aggregate generator dispatch for each scenario. A comparison between model and observed dispatch profiles for selected stations is shown in Fig. 6. Station dispatch was calculated by aggregating DUID output to the station level. For Fig. 5, these values were further aggregated by computing total energy sent out over the 24 h interval under investigation. Each marker within this figure corresponds to a station, with its size indicative of registered capacity. Symmetric log and log-log scales have been used for Figs 4 and 5 respectively to better resolve network flows and generator dispatch values at differing orders of magnitude. Stations that sent no energy out over the interval were arbitrarily assigned a dispatch value of 1 MWh, allowing them to be resolved on a logarithmic scale. Dashed black lines in Figs 4 and 5 have slopes of one, and denote the situation where model and observed values exactly correspond.

Interconnector flow results from Scenario 1 are shown in Fig. 4a and e, while dispatch results are shown in Fig. 5a and d. Strong correspondence is generally observed between model and historic values, with the exception being net aggregate flow over the Victoria - New South Wales (VIC1-NSW1) interconnector for the reduced network. It should be noted that this interconnector is defined as the aggregate flow over several transmission lines, with some entirely residing within New South Wales. When the reduced network is constructed, these internal lines are lost, which likely explains the under representation of energy transfer over this interconnector. Results from Scenario 2 are presented in Fig. 4b and f, which compare model and historic interconnector flows for both the full and reduced networks when generator power injections are fixed. With the exception of VIC1-NSW1 in Fig. 4b and Victoria to South Australia HVDC flows (V-S-MNSP1) in Fig. 4d, model and observed interconnector flows are in close agreement. This suggests that transmission line electrical parameters, interconnector flow limits, and demand allocations are capable of driving network flows that generally correspond well with observed values. Interconnector flow results from Scenario 3 are presented in Fig. 4c,d and g. Aggregate flows between New South Wales and Queensland (NSW1-QLD1 and N-Q-MNSP1) in Fig. 4f corresponded poorly with observed outcomes when using the reduced network and linear cost minimisation objective. Correspondence increases for N-Q-MNSP1 flows when the full network representation is used, shown in Fig. 4g, however large deviations still exist for NSW1-QLD1 flows. Dispatch results for this scenario are shown in Fig. 5b and e. The majority of generators that are observed to be dispatched, based on historic data, are dispatched by the baseline models; however, it can be seen that hydro power plants are systematically over utilised. This is likely due to hydro generators having lower SRMCs relative to thermal power plants, causing the cost minimisation objective functions to favour output from these stations. This may also explain the discrepancy between model and observed interconnector flows for this scenario. Fig. 5c provides a final extension to Scenario 3, where a unit commitment model with ramp-rates, startup-costs, and minimum up and down times is used. Comparing Fig. 5b and c, the tighter clustering of markers around the dashed black line in Fig. 5c suggests that including these additional parameters has a positive effect in terms of emulating the NEM’s operation. Fig. 4h also suggest that the discrepancy between New South Wales and Queensland flows can be reduced when these additional parameters are included. Finally, a comparison of dispatch profiles for several power stations is shown in Fig. 6, with these stations selected based on their ability to illustrate the diversity of outcomes obtained from the benchmark models. For all sub-figures it can be observed that both full and reduced network representations with linear cost minimisation objectives result in similar dispatch profiles. In the case of Bayswater power station, shown in Fig. 6a, the incorporation of additional parameters in the unit commitment formulation reduces the gap between model and observed output. A similar observation can be made for Eraring power station in Fig. 6b. However, it should be noted that this station is under-dispatched for the majority of the interval under investigation, and little evidence of co-movement between model and historic profiles is observed. Co-movements between model and historic dispatch are more pronounced in Fig. 6c, however considerable deviations exist between these values for a majority of hours investigated. For Torrens Island power station, shown in Fig. 6d, results from each scenario suggest the baseline models under dispatch this station between 12 am to 4 pm, but are able to capture peak output occurring between the hours of 4 pm to 10 pm.

### Validation summary

In summary, the Technical Validation procedure first checked the completeness and logical consistency of the generator and network datasets. These procedures revealed that close to all generating capacity that participates in central dispatch is accounted for. The use of an interactive map also assisted in checking relationships between network elements. Regarding the functionality of these data, the assays conducted have demonstrated that feasible models can be constructed. Several scenarios were then developed to investigate the ability of these data to emulate NEM operation using historic dispatch and interconnector flow signals as a basis for comparison. Close correspondence between observed and model interconnector flows and generator dispatch in Scenario 1, with the exception of Victoria - New South Wales interconnector flows for the reduced network, is a positive sign that these data are capable of emulating network operation. Deviations between model and observed interconnector flows for the reduced network also appear to be indicative of a broader trade-off associated with using the reduced network representation. While dispatch results are generally similar for the scenarios investigated, when the full network is used interconnector flows agree more closely with observed outcomes. This is a positive finding, as it suggests the additional information contained within the full network representation is helpful at emulating network flows. Results from Scenario 2 also suggest that network parameters are able to cause power-flows across interconnectors to correspond reasonably well with observed outcomes when power injections are fixed, while dispatch results from Scenario 3 give an indication as to the extent that the relative marginal costs assigned to each generator are capable of capturing dispatch decisions. Large thermal generators generally have close correspondence with observed outcomes, however hydro power plants with relatively low marginal costs are over dispatched, resulting in output from thermal generators to be displaced. Incorporating temporal constraints and startup costs in a unit commitment formulation seems to improve correspondence between model and observed dispatch, suggesting that the inclusion of these parameters is useful with respect to emulating the NEM’s operation. This is further supported by the dispatch profile results shown in Fig. 6. Without the inclusion of these features, model generator dispatch is considerably more flexible than reality. Reducing the flexibility of generator output by including these parameters can help to decrease deviations between model and observed values. The fact that co-movements between model and historic dispatch series can be observed for some stations is another positive sign that these data are able to capture temporal variations in output. In summary, the assays performed demonstrate that feasible models can be constructed from the presented datasets, and that the parameters therein are well suited to emulating basic economic and operational characteristics of the NEM.

## Usage Notes

The derived datasets are made available under a CC BY 4.0 license. For the AEMO and ABS data used, this license is compatible with the AEMO’s copyright permissions^57^, as well as the ABS’s CC BY 4.0 license^58^. Data obtained from GA are also made available under a CC BY 4.0 license, and are necessarily compatible with the licensing approach chosen.

Those seeking to reproduce the results presented are advised to set-up their Python environment using Anaconda, as doing so can simplify the process of installing required packages. For example, Geopandas, used extensively when constructing network and generator datasets, has a number of dependencies. It is convenient to install a Python package, osmnx, via conda-forge as this will handle their installation. A description of all packages and version numbers used in the project are provided in a requirements.txt file within this project’s GitHub repository^42^.

Users seeking more information regarding data available within the MMSDM database are referred to^26^, which provides an overview of each MMSDM table and also describes field names. Descriptions of generator parameters compiled by ACIL Allen, and within the NTNDP database, can be found in^59^. Both documents provide a good reference for those wishing to make use of MMSDM and NTNDP data.

## Additional information

**How to cite this article**: Xenophon, A. & Hill, D. Open grid model of Australiaʼs National Electricity Market allowing backtesting against historic data. *Sci. Data*. 5:180203 doi: 10.1038/sdata.2018.203 (2018).

**Publisher’s note**: Springer Nature remains neutral with regard to jurisdictional claims in published maps and institutional affiliations.

## Supplementary Material



## Figures and Tables

**Table 1:** Total line length by voltage level.

**Voltage [kV]**	**Total line length [km]**
110	4292
132	20768
220	6408
275	11482
330	7449
400	359
500	2170

**Table 2:** System-wide parameters.

**Name**	**Symbol**	**Value**	**Units**
Permittivity of air	*ɛ*	8.854×10^−12^	F/m
System frequency	_*f*_	50	Hz
Base power	*S*_*b*_	100	MVA
Conductor resistance†	*R*	0.168	Ω/km
Conductor geometric mean radius	*D*_*s*_	7.1×10^−3^	m

^†^Values for Pluto AAC 19/3 conductor, obtained from^39^.

**Table 3:** Voltage-dependent electrical parameters.

**V**	*Z*_*base*_^a^	*D*_*min*_^b^	*D*_*eq*_	*L*^c^	*X*_*L*_^d^	*C*_*N*_^e^	*B*_*N*_^f^
[**kV**]	[**−**]	[m]	[m]	[mH/km]	[Ω/km]	[nF/km]	[μS/km]
110	121	1.09	1.64	1.088	0.342	10.02	3.21
132	174	1.25	1.88	1.116	0.350	9.97	3.13
220	484	1.83	2.75	1.191	0.374	9.34	2.93
275	756	2.22	3.33	1.230	0.386	9.05	2.84
330	1089	2.60	3.90	1.262	0.396	8.82	2.77
400	1600	3.07	4.60	1.295	0.407	8.59	2.70
500	2500	3.81	5.71	1.338	0.420	8.31	2.61

^a^$Z_{base}=\frac{V^{2}}{S_{b}},Y_{base}=\frac{1}{Z_{base}}$.

^b^Based on data obtained from^40^.

^c^$L=2\times 10^{-4}\ln\frac{D_{eq}}{D_{s}}$ [H/km].

^d^*X*_*L*_=2π*fL* [Ω/km].

^e^$C_{N}=10^{3}\times \frac{2\pi \varepsilon }{\ln\frac{D_{eq}}{D_{s}}}$ [F/km].

^f^*B*_*N*_=2π*fC*_*N*_ [S/km].

**Table 4:** Network dataset inputs.

**Filename**	Source	Description
ElectricityTransmissionLines_v2/doc.kml	GA^23^	GA transmission line data
ElectricityTransmissionSubstations_v2/doc.kml	GA^24^	GA substation data
MajorPowerStations_v2/doc.kml	GA^25^	GA major power station data
STE06aAUST.shp	ABS^34^	State and territory boundaries
32180 SA2 ERP 2015/doc.kml	ABS^47^	SA2 population data

**Table 5:** Generator dataset inputs.

**Table Name**	Source	Description/contents
DUDETAILSUMMARY	MMSDM^22^	Schedule type and station IDs
DUDETAIL	MMSDM^22^	Registered capacities
STATION	MMSDM^22^	Station names
CO2EII_AVAILABLE_GENERATORS	MMSDM^46^	Emissions intensities
Existing Generators	NTNDP^44^	Technical parameters for units
Coal Cost	NTNDP^44^	Fuel cost profiles for coal units
Gas Cost	NTNDP^44^	Fuel cost profiles for gas units
VOM	NTNDP^44^	VOM costs for each DUID
Heat Rates	NTNDP^44^	DUID heat rates

**Table 6:** Network nodes dataset summary.

**Col.**	Col. Name	Format	Units	Range	Description
1	NODE_ID	str	—	1–960	Node ID
2	STATE_NAME	str	—	—	State in which node is located
3	NEM_REGION	str	—	—	NEM region in which node is located
4	NEM_ZONE	str	—	—	NEM zone in which node is located
5	VOLTAGE_KV	int	kV	110–500	Node voltage
6	RRN	int	—	0–1	If 1 node is a RRN, if 0 node is not a RNN
7	PROP_REG_D	float	—	0.0–0.123	Proportion of NEM regional demand consumed at node
8	LATITUDE	float	N°	−43.2–15.9	Latitude (GDA94)
9	LONGITUDE	float	E°	135.5–153.5	Longitude (GDA94)

**Table 7:** Network edges dataset summary.

**Col.**	Col. Name	Format	Units	Range	Description
1	LINE_ID	str	—	—	Network edge ID
2	NAME	str	—	—	Name of network edge
3	FROM_NODE	int	—	1–960	Node ID for origin node
4	TO_NODE	int	—	1–960	Node ID for destination node
5	R_PU	float	p.u.	6.09×10^−6^–0.407	Per-unit resistance
6	X_PU	float	p.u.	1.52×10^−5^–0.829	Per-unit reactance
7	B_PU	float	p.u.	1.07×10^−5^–1.249	Per-unit susceptance
8	NUM_LINES	int	—	1–4	Number of parallel lines
9	LENGTH_KM	float	km	0.03–315.7	Line length
10	VOLTAGE_KV	float	kV	110–500	Line voltage

**Table 8:** Network HVDC links dataset summary.

**Col.**	Col. Name	Format	Units	Range	Description
1	HVDC_LINK_ID	str	—	—	HVDC link ID
2	FROM_NODE	int	—	605–806	Node ID of origin node
3	TO_NODE	int	—	88–298	Node ID of destination node
4	FORWARD_LIMIT_MW	float	MW	180–594	‘From’ node to ‘To’ node power-flow limit
5	REVERSE_LIMIT_MW	float	MW	180–478	‘To’ node to ‘From’ node power-flow limit
6	VOLTAGE_KV	float	kV	132–400	HVDC link voltage

**Table 9:** AC interconnector locations dataset summary.

**Col.**	Col. Name	Format	Units	Range	Description
1	INTERCONNECTOR_ID	str	—	—	AC interconnector ID
2	FROM_NODE	int	—	40–806	Node ID of origin node
3	FROM_REGION	str	—	—	Region in which ‘From’ node is located
4	TO_NODE	int	—	5–807	Node ID for destination node
5	TO_REGION	str	—	—	Region in which ‘To’ node is located
6	VOLTAGE_KV	float	kV	110–330	Line voltage

**Table 10:** AC interconnector flow limits summary.

**Col.**	Col. Name	Format	Units	Range	Description
1	INTERCONNECTOR_ID	str	—	—	AC interconnector ID
2	FROM_REGION	str	—	—	Region in which ‘From’ node is located
3	TO_REGION	str	—	—	Region in which ‘To’ node is located
4	FORWARD_LIMIT_MW	float	MW	107–1600	‘From’ node to ‘To’ node power-flow limit
5	REVERSE_LIMIT_MW	float	MW	210–1350	‘To’ node to ‘From’ node power-flow limit

**Table 11:** Generator dataset summary.

**Col.**	Col. Name	Format	Units	Range	Description	Source^a^
1	DUID	str	—	—	Unique ID for each unit	^22^
2	STATIONID	str	—	—	ID of station to which DUID belongs	^22^
3	STATIONNAME	str	—	—	Name of station to which DUID belongs	^22^
4	NEM_REGION	str	—	—	Region in which DUID is located	
5	NEM_ZONE	str	—	—	Zone in which DUID is located	
6	NODE	int	—	9–940	Node to which DUID is assigned	
7	FUEL_TYPE	str	—	—	Primary fuel type	^22^
8	FUEL_CAT	str	—	—	Primary fuel category	
9	EMISSIONS	float	tCO_2_/MWh	0.0–1.56	Equivalent CO_2_ emissions intensity	^46^
10	SCHEDULE_TYPE	str	—	—	Schedule type for unit	^22^
11	REG_CAP	float	MW	21–1500	Registered capacity	^22^
12	MIN_GEN	float	MW	0.0–347.2	Minimum dispatchable output	^22,44^
13	RR_STARTUP	float	MW/h	60–1200	Ramp-rate for start-up	^44^
14	RR_SHUTDOWN	float	MW/h	40–9740	Ramp-rate for shut-down	^44^
15	RR_UP	float	MW/h	60–12000	Ramp-rate up when running	^44^
16	RR_DOWN	float	MW/h	60–10080	Ramp-rate down when running	^44^
17	MIN_ON_TIME	int	h	0–16	Minimum on time	^44^
18	MIN_OFF_TIME	int	h	0–16	Minimum off time	^44^
19	SU_COST_COLD	int	$	0–260400	Cold start start-up cost	^44^
20	SU_COST_WARM	int	$	0–89280	Warm start start-up cost	^44^
21	SU_COST_HOT	int	$	0–29760	Hot start start-up cost	^44^
22	VOM	float	$/MWh	0.0–12.5	Variable operations and maintenance costs	^44^
23	HEAT_RATE^b^	float	GJ/MWh	0.0–15.7	Heat rate	^44^
24	NL_FUEL_CONS	float	—	0.0–0.3	No-load fuel consumption as a proportion of full load consumption	^44^
25	FC_2016-17	float	$/GJ	0.0–8.6	Fuel cost for the year 2016–17	^44^
26	SRMC_2016-17	float	$/MWh	0.0–129.7	Short-run marginal cost for the year 2016–17	

^a^Where no source is given, the value has been derived as part of the dataset construction procedure. NEM_REGION and NEM_ZONE were found by determining the region and zone of each generator’s assigned node. FUEL_CAT assigns a generic category to FUEL_TYPE. MIN_GEN was computed by combining minimum output as a proportion of nameplate capacity from^44^ with registered capacities from^22^. SRMC_2016-17 is calculated from VOM, HEAT_RATE, and FC_2016-17 fields, using equation (1).

^b^While not explicitly stated, it is assumed that a lower heating value is referred to. This is consistent with another field in^44^ that gives DUID thermal efficiency in terms of lower heating values.

**Table 12:** Regional demand signals dataset summary.

**Col.**	**Col. Name**	**Format**	**Units**	**Range**	**Description**
1	SETTLEMENTDATE	timestamp	—	1/6/2017 12:30:00 AM–1/7/2017 12:00:00 AM	Trading interval
2	NSW1	float	MW	6298.7–11652.8	New South Wales demand signal
3	QLD1	float	MW	4864.0–7728.7	Queensland demand signal
4	SA1	float	MW	1002.9–2287.1	South Australia demand signal
5	TAS1	float	MW	921.0–1708.6	Tasmania demand signal
6	VIC1	float	MW	3795.8–7357.3	Victoria demand signal

**Table 13:** DUID dispatch profiles.

**Col.**	**Col. Name**	**Format**	**Units**	**Range**	Description
1	SETTLEMENTDATE	timestamp	—	1/6/2017 12:30:00 AM–1/7/2017 12:00:00 AM	Trading interval
2–265	(DUID)	float	MW	—	DUID dispatch profile
^a^Columns correspond to DUIDs.					

**Table 14:** Installed capacity comparison between generator dataset and AEMO generation information for June 2017.

	**Gen. Dataset**	**AEMO Gen. Info.**	**Difference**	
	**(MW)**	**(MW)**	**(MW)**	**%**
			**New South Wales**	
Coal	10240	10160	80	0.78
Hydro	4054	2525	1529	37.72
OCGT/CCGT	2038	2029	9	0.45
Solar	212	211	1	0.47
Wind	655	480	175	26.72
			**Queensland**	
Coal	8119	8186	−67	−0.83
Hydro	644	652	−8	−1.30
OCGT/CCGT	3440	3297	143	4.16
			**South Australia**	
OCGT/CCGT	2933	2981	−48	−1.64
Wind	1309	1207	102	7.77
			Tasmania	
Hydro	2171	2170	1	0.04
**OCGT/CCGT**	371	386	−15	−4.04
Wind	168	168	0	0.00
			Victoria	
Coal	4690	4630	60	1.28
Hydro	632	2213	−1581	−250.16
**OCGT/CCGT**	2374	2382	−80	−0.34
Wind	965	965	0	0.00
**Total**	**45015**	**44643**	**372**	**0.83**
